# Pseudoephedrine—Benefits and Risks

**DOI:** 10.3390/ijms22105146

**Published:** 2021-05-13

**Authors:** Krystyna Głowacka, Anna Wiela-Hojeńska

**Affiliations:** Department of Clinical Pharmacology, Wroclaw Medical University, Borowska 211a St., 50-556 Wroclaw, Poland; anna.wiela-hojenska@umed.wroc.pl

**Keywords:** pseudoephedrine, sympathomimetic, adverse reactions, non-medical use

## Abstract

Pseudoephedrine (PSE) is a drug with a long history of medical use; it is helpful in treating symptoms of the common cold and flu, sinusitis, asthma, and bronchitis. Due to its central nervous system (CNS) stimulant properties and structural similarity to amphetamine, it is also used for non-medical purposes. The substance is taken as an appetite reducer, an agent which eliminates drowsiness and fatigue, to improve concentration and as a doping agent. Due to its easier availability, it is sometimes used as a substitute for amphetamine or methamphetamine. Pseudoephedrine is also a substrate (precursor) used in the production of these drugs. Time will tell whether legal restrictions on the sale of this drug will reduce the scale of the problem associated with its misuse.

## 1. Introduction

Pseudoephedrine (PSE) and ephedrine (E) are alkaloids derived from various species of *Ephedra spp*. of the Ephedraceae family. The most common source of their extraction is *Ephedra sinica*, also known as Ma Huang. The history of the use of Ephedra products in medicine is very long; they have been used in China for over 5000 years and in the Middle East for over 2000 years in the treatment of bronchial asthma, fever, coughs and colds, hay fever, oedema, bronchitis, urticaria, chronic hypotension, and rheumatism. Nowadays, they are also used as stimulants, the so-called energisers, and as agents reducing appetite, body weight and increasing energy consumption. They are popular with bodybuilders, athletes, schoolchildren and students [1,2,3,4,5,6].

Although PSE is an E stereoisomer, it has weaker vasoconstrictive effects and smaller effects on the central nervous system (CNS) compared to ephedrine. Pseudoephedrine is one of the four stereoisomers of ephedrine (from the natural alkaloid Ephedra from China or India), because, due to having two stereogenic carbon atoms, it exists as four different diastereoisomers. Synthetically obtained compounds of PSE occur in the form of a racemate of diastereoisomers, whose action is twice as weak compared to compounds of natural origin. This is easily explained, because PSE contains only one active diastereoisomer, while the second one is as a ballast [2,7,8,9,10].

## 2. Mechanism of Action

Pseudoephedrine is a sympathomimetic with a mixed mechanism of action, direct and indirect. It indirectly stimulates alpha-adrenergic receptors, causing the release of endogenous norepinephrine (NE) from the granularity of neurons, while it directly stimulates beta-adrenergic receptors [11,12,13].

**Figure 1:**
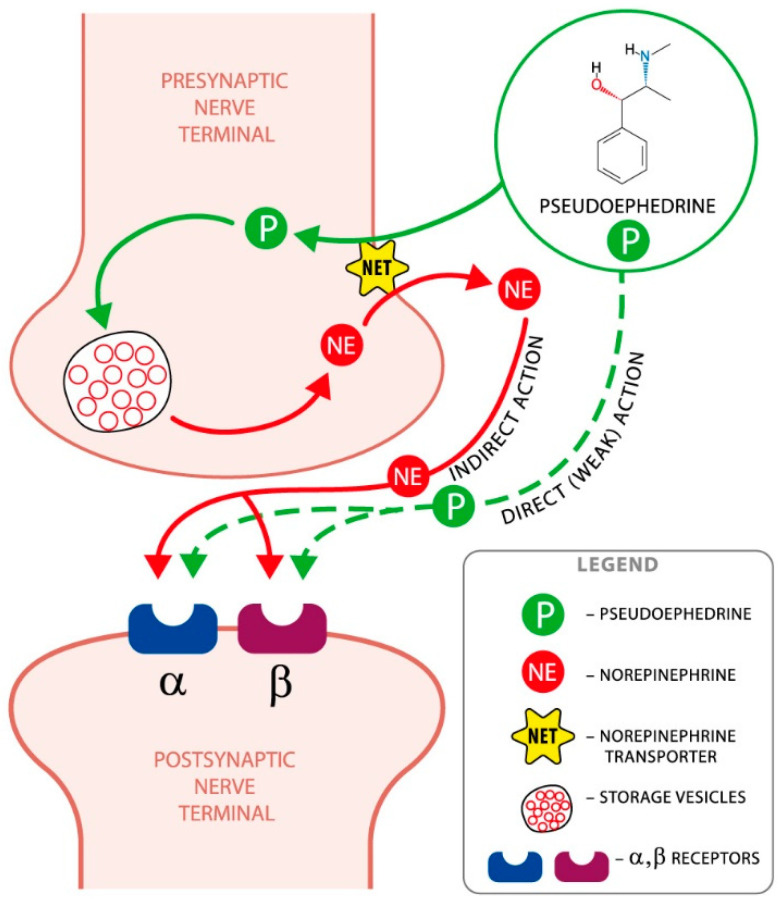
The mechanism of pseudoephedrine action. The principal mechanism by which pseudoephedrine achieves its effects is by displacing the norepinephrine (noradrenaline) from the storage vesicles in the presynaptic neurons; then, it is released into the neuronal synapse and becomes available to activate the alpha and beta postsynaptic adrenergic receptors.

It has an effect similar to ephedrine, but slightly weaker, and has a lower ability to induce tachycardia and increase systolic blood pressure. Its central effect is weaker than that of amphetamine, and its peripheral effect is similar to that of epinephrine [9]. The mechanism of pseudoephedrine action is shown graphically in Figure 1.

## 3. Pharmacokinetics

Unlike epinephrine and norepinephrine, pseudoephedrine is active after oral administration and is easily absorbed from the gastrointestinal tract. The onset of action occurs after 30 min and after 1–4 h the drug reaches its maximum concentration in the blood. When using the extended-release formulation, this time is twice as long. PSE is mainly excreted unchanged in the urine (43–96%); only a small amount, approximately 1–6%, is metabolised in the liver by N-demethylation to the active metabolite norpseudoephedrine (cathine). The time the drug remains in the body depends on the pH of the urine; the value of the biological half-life (*t*_0.5_) decreases when the urine is acidic, and increases when the urine is alkaline [8,14,15,16,17,18,19]. Selected pharmacokinetic properties of pseudoephedrine are presented in Table 1.

## 4. Special Risk Populations

PSE is present in numerous over-the-counter preparations and is taken by pregnant women. According to the US Food and Drug Administration (FDA), the drug belongs to category C, which means that animal studies have shown adverse effects on the foetus, although there are no controlled studies in pregnant women. It can therefore only be used in cases where the benefit to the mother outweighs the potential risk to the foetus [18]. Although there is insufficient evidence of a teratogenic effect of pseudoephedrine, the results of some studies suggest that it should be used with caution. It has been found that the use of preparations containing this compound in the first trimester of pregnancy may increase—almost twice (1.8 times) compared to the control group—the risk of congenital evisceration (a developmental defect of the abdominal wall with displacement of the intestines outside the abdominal cavity). However, these observations were made in women using mainly combined preparations, so the effect of other ingredients cannot be excluded. In the third trimester of pregnancy, PSE may cause reduced blood flow in the uteroplacental circulation, especially in women who smoke [20]. Another randomised population-based study conducted in Massachusetts, USA, involving 3271 live-born children without malformations, assessed the risk of preterm birth in relation to the use of decongestants in the upper respiratory tract. The population-based retrospective cohort study was conducted between 1998 and 2008 on the basis of an interview. It was found that the use of pseudoephedrine in the second and third trimester of pregnancy for asthma, rhinitis, colds, nasal and sinus congestion, reduces the risk of preterm birth compared to women who have not used these drugs. These observations, however, had many limitations because the women participating in the study constituted a highly heterogeneous group in terms of age, race, education, social position, economic conditions, health status, and use of stimulants [21].

During lactation only small amounts, about 0.5% of a single oral daily dose, pass into breast milk. However, even a single dose of 60 mg reduces daily milk production by 24%.

There are no conclusive results of studies on the efficacy and safety of PSE preparations in children. Most reports warn against their use in the therapy of children under 12 years of age, although a multicentre, double-blind, placebo-controlled randomised study by Gelotte et al. has shown the efficacy and safety of pseudoephedrine hydrochloride 30-mg tablets in children from 6 to 11 years of age in order to temporarily relieve nasal congestion caused by colds [22].

The effects of PSE in elderly patients have not been specifically investigated. It is recommended to follow the adult dosage regimen, with special attention to kidney and liver function. If these organs are severely impaired, the drug should be used with caution. Overdosing in people over 60 years of age may cause hallucinations, CNS depression, seizures and death [18,19].

## 5. Consequences of Pseudoephedrine Use

The drug reduces congestion of the upper respiratory tract mucosa, especially in the nose and paranasal sinuses (after oral administration), which in turn reduces the swelling, the amount of secretions and clears the nose. The sympathomimetic effect of pseudoephedrine may also improve the patency of the Eustachian tube and equalise the pressure in the middle ear during changes in atmospheric pressure while diving or flying by plane. The administration of 120 mg of pseudoephedrine to an adult at least 30 min before a flight may reduce earache. However, no similar effect has been observed in children. Pseudoephedrine is also effective in cases of urinary incontinence [9,23].

Similarly to other sympathomimetics, PSE stimulates the sympathetic system to fight-or-flight reactions—speeds up breathing, increases blood pressure, accelerates heart rate, narrows peripheral blood vessels, causes bronchodilatation, increases blood glucose levels, stimulates the CNS, as well as giving a sense of an energy surge and improving mood [9,24].

## 6. Clinical Use and Contraindications

Pseudoephedrine is recommended for the symptomatic treatment of obstruction in the nasal cavity, paranasal sinuses and the Eustachian tube. Other indications include vasomotor rhinitis and adjunctive therapy in allergic rhinitis and otitis media [9,18,19].

Contraindications to pseudoephedrine use are hypersensitivity to the drug, cardiovascular diseases (hypertension and coronary artery disease), impaired function of organs responsible for elimination of the drug (severe liver dysfunction, moderate or severe renal dysfunction), hyperthyroidism, narrow-angle glaucoma, benign prostatic hyperplasia, diabetes mellitus, mental agitation and treatment with monoamine oxidase inhibitors (MAO inhibitors) currently or in the last two weeks. Contraindications also include physiological conditions such as pregnancy and lactation, and age under 2 years. The extended-release form of the drug should not be used until the age of 12 years [18,19].

Although there are no reports of pseudoephedrine disturbing psychophysical performance, people driving motor vehicles should exercise caution and not use doses higher than recommended.

Studies evaluating the effect of this drug on daytime sleepiness and fatigue in patients suffering from perennial allergic rhinitis showed no positive or negative effect compared to placebo [25].

## 7. Dosage

Pseudoephedrine is found as a hydrochloride or sulphate in doses ranging from 30 to 120 mg in about 30 medicinal products. In combined preparations, it is most often compounded with antihistamines, analgesics and antitussive drugs; it comes in the form of plain, coated or extended-release tablets, capsules, syrup, powder or granules for oral fluid preparation. The recommended dosage for adults is 60 mg 3–4 times a day or in the extended-release form—120 mg every 12 h. In children the dose is 1 mg/kg body weight 4 times a day. In Poland, there are many products containing pseudoephedrine with its content varying from 30 to 120 mg [15,18,19].

## 8. Overdose

The maximum permissible daily dose of pseudoephedrine is 240 mg for an adult, 120 mg for children aged 6–12 years and 60 mg for children aged 2–5 years [18,19]. Toxic effects may appear not only during the use of increased doses of the drug, but also in people who are particularly sensitive to the effects of sympathomimetics. Prolonged use of PSE, especially at short intervals, may reduce the effectiveness of the drug (tachyphylaxis) and increase the risk of toxic effects. As the result of an overdose, the symptoms of a sympathomimetic effect may vary. Sometimes there is a depressive effect on the CNS (sedative effect, apnoea, decreased ability to concentrate, cyanosis, coma and circulatory collapse), other times a stimulating effect (insomnia, hallucinations, tremors and convulsions). In extreme cases, death may occur. Symptoms of overdose also include headache, dizziness, anxiety, euphoria, tinnitus, blurred vision, ataxia, chest pain, tachycardia, palpitations, increased or decreased blood pressure, increased thirst, sweating, difficulty urinating, nausea and vomiting. In children, more frequently observed symptoms are dry mouth, wide and rigid pupils, hot flushes, fever, and digestive tract dysfunctions [9,18,19].

## 9. Adverse Reactions

Pharmacotherapy is inevitably associated with the risk of drug-related complications; the most controversial is the effect of pseudoephedrine on blood pressure and its consequences. Some literature data suggest that oral sympathomimetic drugs may dangerously increase blood pressure, while others reassure that the danger is exaggerated. One meta-analysis of randomised controlled trials showed that PSE at the recommended doses had no effect on systolic and diastolic blood pressure in healthy or controlled hypertensive patients. Systolic blood pressure increases by an average of 1 mm Hg and the heart rate increases by three beats per minute. Only about 3% of the analysed patients had pressures above 140/90 mm Hg [26]. During the use of pseudoephedrine preparations, reported cases include an acute coronary syndrome in a patient who performed hard physical work and smoked for 30 years; a myocardial infarction, hypertensive crisis and NSTEMI (Non-ST-Segment Elevation Myocardial Infarction) without ST segment elevation after taking the drug in the extended-release form by an 87-year-old man with history of mild dementia, glaucoma and atrial fibrillation; as well as an increased blood pressure of 220/140 mm Hg, hyperglycaemia, haemorrhagic stroke, strong, reversible spasm of blood vessels and tachycardia in a driver working at night, a nicotine addict and concomitant drug user for more than 20 years [15,26,27,28,29]. The effect of pseudoephedrine may induce non-convulsive epileptic states in predisposed individuals with pre-existing neurological disorders [15,30]. The risk of complications increases in patients with impaired renal and hepatic function. Unusual behaviour and myoclonic convulsions were observed in a 64-year-old man suffering from renal failure, who took 240 mg of PSE daily to treat rhinitis [15]. Severe agitation and disorientation can be expected in patients with phenylketonuria due to a disturbed metabolism of catecholamines [9]. Adverse effects of PSE can occur with both oral and intranasal administration after a single dose or after prolonged (5 days) treatment, without affecting the dose and irrespective of the vascular condition and age. A 2003 French study analysed adverse events with intranasal decongestants reported to regional pharmacovigilance centres by healthcare professionals. There were 22 episodes of arterial hypertension, 15 cases of convulsions and 4 cases of stroke after oral administration of drugs containing pseudoephedrine [6]. It can also induce ischemic colitis when used for as little as 3 days or up to 2 years, in a dose range of 60 to 900 mg/day [31]. Less common adverse effects are skin reactions—cases of scarlet fever-like rash, erythematous spots, skin exfoliation of the palms and soles of the feet, and Baboon syndrome, clinically manifested by a rash mainly on the buttocks and within the larger folds of skin, have been reported [32,33]. When used in therapeutic doses, PSE may be responsible, especially in children, for the occurrence of pain and dizziness, increased heart rate, excessive agitation, insomnia and hallucinations [9]. “Parasitic” hallucinoses (attacking spiders and insects) have been observed in children after taking an OTC (over-the-counter) drug containing pseudoephedrine and triprolidine to treat inflammation of the nasal mucosa [9,15]. In 2007 Wingert et al. detected pseudoephedrine in a postmortem analysis of 13 unexpected deaths of children under 2 years of age taking cold medications in the Philadelphia region. Similar observations were made in 2008 by Rimsza and Newberry, who reviewed case files of unexpected deaths of children taking cold medications. PSE preparations should not be used in patients before the age of 12, and according to the French Society of Otorhinolaryngology, until the age of 15 [6]. On the pharmaceutical market, however, there are preparations allowing their administration to younger patients, e.g., from 7 years of age. The addictive potential of PSE is confirmed by the case of a 37-year-old woman who abused it for its euphoric effect, increasing the doses over five years, using 3000–4500 mg daily. Sudden discontinuation of the drug resulted in depressed mood, visual hallucinations and a feeling of fatigue [15]. Table 2 presents the adverse effects of pseudoephedrine and the incidence of some of them [18,19,34,35].

## 10. Interactions

When several drugs are used concomitantly, an interaction may occur between them, as a result of which the final effect of some drugs changes. The combination of pseudoephedrine with other sympathomimetic drugs and monoamine oxidase inhibitors (MAO) should be avoided. Inhibition of intra-neuronal NE breakdown in sympathetic nerves by MAO inhibitors leads to an increase in the amount of neuromediator released by pseudoephedrine, which may lead to hypertensive crisis and bradycardia. Due to the long duration of action of MAO inhibitors, a 14-day interval between taking the drugs should be maintained. Due to the possibility of vasoconstriction and an increase in blood pressure, especially in patients at risk of ischemic stroke, the concomitant use of pseudoephedrine with vasoconstrictor drugs such as ergotamine, dihydroergotamine, linezolid, oxytocin, ephedrine, phenylephrine and bromocriptine is not recommended. Pseudoephedrine may also increase myocardial excitability and affect ventricular rhythm, especially in patients with cardiac diseases who are hypersensitive to the cardiac effects of sympathomimetics. Combination with caffeine present in many OTC medications, dietary supplements and energy drinks may cause hyperglycaemia and an increase in body temperature. Pseudoephedrine used concomitantly with the drugs listed in Table 3 may cause undesirable interactions, resulting in the weakening or potentiation of the drug effects [18,19,36].

**Figure 2:**
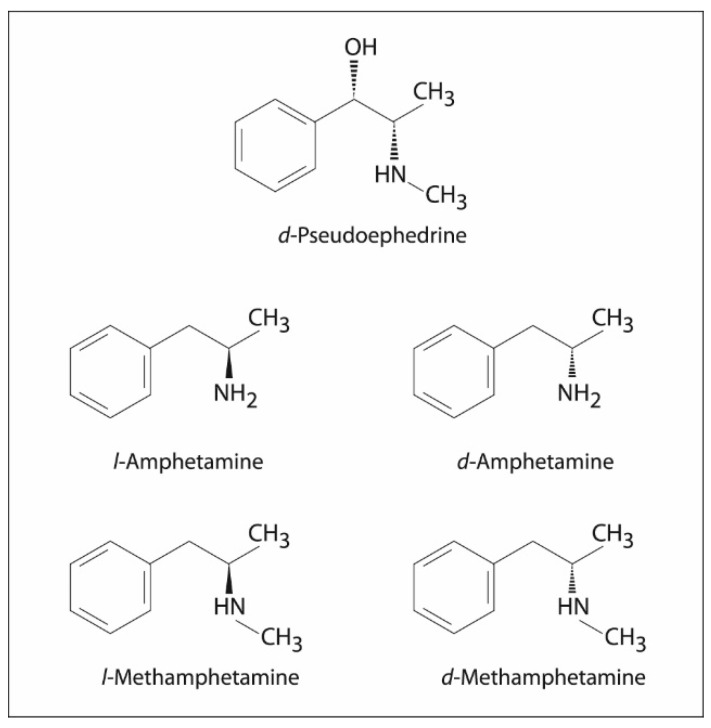
Chemical structure of pseudoephedrine and optical isomers of amphetamine and methamphetamine.

Pseudoephedrine may also be responsible for a false-positive urine test for amphetamine and methamphetamine. The structural similarity to these drugs means that they may cross-react in a test using the immunological method. Figure 2 shows the similarity in chemical structure of pseudoephedrine, amphetamine and methamphetamine [3,37,38].

## 11. Self-Medication

The widespread availability of drugs, especially those sold over the counter, and their advertising encourages many people to self-medicate, also with products containing pseudoephedrine. Simultaneously using several products under different names for rhinitis, sinusitis, cold or allergic rhinitis—the patient, unaware, exposes himself to overdose. Moreover, as shown by the study by Pawlaczyk et al., a large percentage of people use PSE preparations without consulting a doctor. The most frequently reported adverse reactions by patients included CNS disturbances—agitation, insomnia, sedation, headache. It should be noted that currently 720 mg of the active substance can be purchased at a time at a pharmacy open to the public—1 package containing 12 tablets of 60 mg. The legislator justified the above regulation with the statement that such an amount will allow for an effective and safe self-treatment. A patient who purchases a drug at a pharmacy has the opportunity to consult a pharmacist [15]. Unfortunately, as demonstrated by the study by Gołda et al., the quality of a pharmaceutical consultation regarding the expedition of pseudoephedrine at a dose of 60 mg does not always ensure safety. It is therefore necessary to develop and implement specific procedures in this regard [39].

## 12. Non-Medical Use

Medicinal products are not always used as intended. Pseudoephedrine—due to its properties including increased muscle contractility, increased blood flow to skeletal muscles, stimulation of glycogenesis, bronchodilatation, increased cardiac tropisms, activation of the central nervous system, suppression of appetite—is also used as a slimming agent and in sport—as an ergogenic agent, i.e., improving efficiency, allowing for faster regeneration and better performance. The influence of PSE on sporting performance has long been a subject of debate, and observations do not always confirm this effect; however, it is on the list of substances prohibited for use by athletes during competitions. Due to its wide availability, it is considered an anti-doping rule violation when its concentration in urine exceeds 150 μg/mL. This list is a mandatory international standard and is updated annually by the World Anti-Doping Agency (WADA), which is part of the World Anti-Doping Programme. The current list has been in effect since 1 January 2021 [12,13,40,41].

Increased interest in preparations containing pseudoephedrine is related to its use for recreational purposes, especially by adolescents and young adults, as well as for the production of psychoactive substances—the synthesis of methamphetamine and methcathinone (ephedrone), used as designer drugs [42,43]. Until recently, publicly available drugs with PSE were sold on the Polish market without restrictions; this demand was exploited by some pharmacies. It happened despite the alarm of pharmaceutical inspectors. The territorial-quantitative analysis of sales data, prepared by US Pharmacia in cooperation with the Main Pharmaceutical Inspector and the National Bureau for Drug Prevention in 2009 and 2010, allowed for an indirect assessment of the scale of the phenomenon of non-medical use of medicines containing pseudoephedrine. These data show that above-average sales of preparations containing PSE were territorially diversified. A total of 29 areas with intensified sales and 3 medicinal products (Cirrus, Acatar AT, Sudafed) were identified. The main Polish regions with intensified sales of these products were the four south-western border provinces. High intensification of the problem was observed in Lower Silesia (757,000 packages in 2009 and 1332,000 packages in the period from January to September 2010). Most of the pseudoephedrine available in medicinal products ended up in the Czech Republic, where it was used as a precursor to synthesise a very popular in the country local methamphetamine called Pervitin [44].

For many years, the Institute of Forensic Expertise in Kraków (Poland) has been conducting studies on manufacturing psychoactive substances from medicines. People experimenting with home drug production often use PSE preparations for this purpose, in Poland most frequently Sudafed and Acatar. The method is based on the oxidation of PSE with large amounts of potassium permanganate in an acidified environment; the resulting ephedrone solution also contains large amounts of manganese. After intravenous injection, it quickly enters the CNS, accumulating in subcortical structures, and has a strong neurotoxic effect. Frequently underestimated postural and speech disorders as symptoms of manganese encephalopathy are observed after only 5–9 months. They manifest as difficulties in maintaining balance with a tendency to fall backwards, problems with standing up without support or assistance and moving backwards. Typical is the so-called “cock gait” caused by dystonic contraction of the foot and calf muscles. Speech disturbances, sometimes making contact with the environment difficult or impossible, occur in the form of muffled speech and palilalia; they take the form of dysarthria. Compulsive laughter is not uncommon. Some people develop an extrapyramidal syndrome, including symptoms such as slowness of movement, bradykinesia, muscle stiffness, hypomimia, micrography (reduced handwriting), and difficulty with fine movements. Rest tremor observed in Parkinson’s disease is rare. The diagnosis of manganese encephalopathy requires confirmation of intravenous use of home-prepared ephedrone with potassium permanganate; this is followed by a typical magnetic resonance imaging of the brain, which shows hyperintense lesions in the globus pallidus, shells and thalamic nuclei in the T1 sequence. The differential diagnosis with Parkinson’s disease highlights insensitivity or minimal sensitivity to anti-Parkinsonian drugs, including L-dopa preparations [45,46,47,48]. Atypical pneumonia, phlebitis, limb necrosis, hepatic and pancreatic disorders have also occurred in people taking Sudafed products [9,44].

The Regulation of the Minister of Health in Poland on the list of substances with psychoactive effects and the maximum level of their content in a medicinal product, in force since 1 January 2017, constituting a restriction on the dispensing of medicinal products as part of a single sale, has not solved the problem of abuse of medicines containing psychoactive substances, including the purchase of medicinal products in bulk by some people for non-medical use of pseudoephedrine as a precursor or a psychoactive substance. This has made access to medicines with pseudoephedrine somewhat more difficult, but not impossible. Perhaps changing the availability category of medicinal products containing pseudoephedrine from ‘OTC’ to ‘Rx’ and selling them on medical prescription would be a more effective solution.

## Figures and Tables

**Table 1:** Pseudoephedrine pharmacokinetics.

Pharmacokinetic Parameters of Pseudoephedrine	
Onset of action	30 min
Time to reach C_max_	1–4 h
Time to reach C_max_ after administration of the extended-release formulation	2–6 h
Duration of action	4–12 h
Distribution coefficient	2.64–3.51 l/kg
Biological half-life	3–16 h
Renal clearance	0.44–0.46 l/h/kg, 7.3–7.7 mL/min/kg

**Table 2:** Complications after the use of pseudoephedrine.

Pseudoephedrine Adverse Effects
CNS stimulation—sleep disturbances (>30%), anxiety, headache, muscle tremor, confusion
Dryness of mucous membranes of the mouth, nose and throat (>15%)
Digestive tract dysfunction—indigestion, nausea, vomiting, decreased appetite, irritation of the gastric mucosa (5%)
Cardiac arrhythmias, tachycardia, increased blood pressure
Excessive sweating, hyperglycemia, urination disorders
Allergic reactions—redness, rashes
Psychological dependence

**Table 3:** Adverse drug interactions of pseudoephedrine.

Pseudoephedrine Interactions	
Other Concomitantly Used Medicines and Substances	Type and Consequence of Interaction
Antacids (e.g., aluminium hydroxide), proton pump inhibitors	Increase in PSE absorption rate
Kaolin clay	Decreased rate of absorption of PSE due to its adsorption on the surface of kaolin clay
Digitalis glycosides	Increased ectopic activity of the heart’s conducting system, arrhythmia
MAO inhibitors (phenelzine, selegiline, tranylcypromine, procarbazine)	Synergistic sympathomimetic effect, significant increase in blood pressure, hypertensive crisis, bradycardia— 14-day interval between drugs is required
Tricyclic antidepressants	Increased effect of PSE, increased risk of hypertension and cardiac arrhythmias—concomitant use is not recommended
Methyldopa, guanethidine, reserpine	PSE reduces the antihypertensive effect in addition to the drugs—concomitant use is not recommended
Appetite suppressants	Risk of increased blood pressure, increased heart rate—concomitant use is not recommended
Ergotamine, dihydroergotamine, linezolid, oxytocin, ephedrine, phenylephrine, bromocriptine	Risk of vasoconstriction and increase in blood pressure—concomitant use is not recommended
Urine alkalinisation (e.g., sodium bicarbonate)	Urine alkalinisation increases the reabsorption of PSE, the risk of seizures, anxiety, restlessness, insomnia, tachycardia
Inhalation agents for general anaesthesia	Acute hypertensive reaction in the perioperative period—discontinuation of PSE is recommended 24 h before the planned general anaesthesia
Caffeine	Elevated body temperature, hyperglycaemia, insulinaemia, increased C-peptide levels
Ethyl alcohol	Acute psychosis
